# GLP-1 Receptor Agonists in Periodontology: Mechanisms, Clinical Evidence, and Implications for Care

**DOI:** 10.3390/biom16060857

**Published:** 2026-06-11

**Authors:** Irina-Georgeta Sufaru, Bogdan Constantin Vasiliu, Monica Hancianu, Stefan-Ioan Stratul, Monica Silvia Tatarciuc, Gianina Iovan, Diana Tatarciuc, Ioana Rudnic, Diana Hanu, Sorina Paduraru, Sorina Mihaela Solomon

**Affiliations:** 1 Grigore T. Popa University of Medicine and Pharmacy, 700115 Iasi, Romania; ursarescu.irina@umfiasi.ro (I.-G.S.); monica.hancianu@umfiasi.ro (M.H.); monica.tatarciuc@umfiasi.ro (M.S.T.); gianina.iovan@umfiasi.ro (G.I.); diana.tatarciuc@umfiasi.ro (D.T.); ioana.rudnic@umfiasi.ro (I.R.); diana.roman@umfiasi.ro (D.H.); sorina.paduraru@umfiasi.ro (S.P.); sorina.solomon@umfiasi.ro (S.M.S.); 2 Department of Periodontology, Faculty of Dental Medicine, Anton Sculean Research Center for Periodontal and Peri-Implant Diseases, “Victor Babes” University of Medicine and Pharmacy, 300041 Timisoara, Romania; stratul.stefan@umft.ro

**Keywords:** GLP-1 receptor agonist, periodontitis, peri-implantitis, dipeptidyl peptidase-4 (DPP-4), periodontal ligament stem cells, osteoimmunology

## Abstract

GLP-1 receptor agonists (GLP-1RAs) are widely used in the treatment of type 2 diabetes and obesity and are increasingly relevant in periodontal and implant practice. This review covers mechanisms, preclinical and early human evidence, and practical periodontal considerations; the structured database search is conducted in accordance with the Scale for the Assessment of Narrative Review Articles (SANRA) and the International Committee of Medical Journal Editors (ICMJE) principles. Two pathways explain GLP-1RAs’ relevance: indirect effects from better glycemic control, weight loss, and reduced inflammation; and direct tissue effects involving GLP-1R signaling and the GLP-1/dipeptidyl peptidase-4 (DPP-4) axis. Preclinical studies show reduced inflammation, osteoclast activity, and alveolar bone loss, along with improved periodontal stem cell function under hyperglycemia or inflammation via Nuclear Factor-kappaB (NF-kappaB), Wingless-related integration site (Wnt)/beta-catenin, and Mitogen-Activated Protein Kinase (MAPK) pathways. Animal studies on implants and local delivery, including exendin-4 platforms, suggest osteometabolic benefits. Human data are limited and mostly observational, and confounders include metabolic status, smoking, medication, and nutrition. Oral side effects such as xerostomia and dehydration are also noted. At present, GLP-1RA therapy should be regarded as a contextual modifier of periodontal risk and healing capacity rather than as a stand-alone periodontal therapy.

## 1. Introduction

Glucagon-like peptide-1 receptor agonists (GLP-1RAs) have evolved from being primarily a diabetology-focused treatment class into an important component of modern metabolic medicine. Their use has rapidly expanded in both type 2 diabetes (T2D) [1] and obesity [2], now including not only established agents such as liraglutide and semaglutide, but also newer dual incretin agonists such as tirzepatide. Importantly, approved and emerging indications for GLP-1RAs now extend beyond glycemic and weight management to include cardiovascular risk reduction in overweight or obese adults with established cardiovascular disease; chronic kidney disease in patients with T2D; metabolic dysfunction-associated steatohepatitis (MASH); and obstructive sleep apnea [1,3]. In parallel, while the core diagnostic criteria for T2D have remained stable, recent updates to the American Diabetes Association (ADA) Standards of Care emphasize broader screening for prediabetes and T2D risk, as well as improved assessment of body composition for obesity [4]. Together, these developments mean that dental and periodontal clinicians are increasingly treating patients who are on incretin pathway therapy for a widening range of metabolic and systemic indications [5], many of whom may not carry a diabetes diagnosis.

This shift is highly relevant to periodontology. Periodontists and implant clinicians are now treating more patients who use GLP-1RAs for T2D, obesity, or both, often within complex multi-drug metabolic regimens. In such cases, periodontal inflammation, wound healing, maintenance behavior, and peri-implant tissue stability may be affected not only by the underlying metabolic disorder but also by the systemic and tissue-level effects of GLP-1RA therapy [6]. Recent reviews focusing on periodontal health reflect this evolving clinical landscape and have started to synthesize preclinical and early clinical evidence linking GLP-1 pathways to periodontal and peri-implant outcomes [6,7,8].

A major reason this topic deserves careful appraisal is that any potential periodontal benefit may result from at least two overlapping mechanisms. One is direct, involving receptor-mediated effects on inflammatory signaling, bone remodeling, and regenerative processes within periodontal and peri-implant tissues; the other is indirect, where improved glycemic control, weight loss, and reduced systemic inflammation create a healthier host environment that favors periodontal stability and treatment response [9]. Differentiating between these pathways is crucial for responsibly interpreting the literature and for avoiding overstating drug effects specific to periodontal health.

Interest in GLP-1RAs in periodontology stems from the well-established oral–systemic connection among periodontitis, diabetes, and obesity. The link between periodontitis and diabetes is bidirectional: diabetes increases the risk of periodontal inflammation and tissue damage, while periodontitis adds to the systemic inflammatory load and is linked to poorer glycemic control and less favorable diabetes outcomes [10,11,12,13,14,15,16]. This idea has been consistently supported by consensus and epidemiological studies and remains central to periodontal medicine.

A similarly bidirectional relationship is increasingly recognized between periodontitis and obesity. Obesity is associated with chronic low-grade inflammation, dysregulated adipokine signaling, and altered immune function, all of which may intensify periodontal inflammation and impair treatment response [17,18,19,20,21]. Conversely, periodontitis may contribute to systemic inflammatory stress and metabolic dysfunction, reinforcing the obesity-periodontitis connection as an important oral–systemic pathway [22].

An additional mechanism linking obesity to periodontal inflammation involves lipid-mediated activation of innate immune signaling. In obese individuals, elevated circulating free fatty acids, particularly saturated fatty acids, can activate Toll-like receptor 2 (TLR2) and TLR4 signaling pathways, leading to NF-κB-dependent pro-inflammatory cytokine production in monocytes and macrophages [23,24]. TLR2 and TLR4 are also key pattern recognition receptors in the periodontal host response, mediating innate immune activation by periodontopathic bacteria and their products, including lipopolysaccharide [25]. In obese patients with periodontitis, these pathways may converge: metabolic lipid overload and microbial challenge could synergistically amplify gingival inflammation and alveolar bone resorption through shared TLR-dependent signaling. This is particularly relevant to GLP-1RA research because recent evidence shows that central GLP-1R activation inhibits TLR agonist-induced inflammation independently of metabolic improvement [26], suggesting a potential mechanistic pathway by which GLP-1RAs could attenuate lipid-driven innate immune amplification in periodontitis.

This context is especially relevant for GLP-1RA research because many patients now take these drugs for obesity without diabetes, creating a clinical group whose periodontal implications might differ from those of traditional diabetic patients [8].

This narrative review offers a critical, clinically focused assessment of the importance of GLP-1RA therapy in periodontology and implant dentistry. It combines mechanistic, preclinical, and early clinical evidence while clearly distinguishing between biological plausibility and clinically proven effects.

The specific aims, aligned with the concept blocks used in the search strategy, are as follows: (1) to review the pharmacology and pleiotropic actions of GLP-1RAs relevant to periodontal biology, including the GLP-1/DPP-4 axis and its intersection with host–microbe interactions; (2) to synthesize mechanistic and regenerative evidence, including inflammatory, osteometabolic, and PDLSC-related pathways, with attention to translational relevance and confounding factors; (3) to critically evaluate the available preclinical and human evidence on periodontal and peri-implant outcomes, including alveolar bone remodeling, gingival inflammation, and peri-implantitis; and (4) to discuss practical clinical implications, oral adverse effects, and priorities for future research, including endpoint standardization and integration into interdisciplinary care.

## 2. Materials and Methods

This article was developed as a narrative review with a structured search strategy and a framework for critical synthesis. The goal was to provide a clinically relevant and conceptually integrated overview of GLP-1RAs in periodontology. A narrative approach was selected because the available literature is highly diverse, including in vitro studies, animal models, observational human studies, early clinical reports, and contextual reviews. In these conditions, a traditional systematic review centered on a single outcome would be difficult to perform and have limited interpretive value.

The review was structured according to SANRA (Scale for the Assessment of Narrative Review Articles). Relevant ICMJE (International Committee of Medical Journal Editors) guidance for responsible manuscript preparation, source attribution, and citation practice was also followed, especially the emphasis on accurate referencing and, when possible, direct citation of primary sources [27].

A thorough literature search was conducted across PubMed/MEDLINE, Scopus, Embase, and Web of Science to identify publications on GLP-1RA use and periodontal or peri-implant biology. The search aimed to include both basic incretin biology and current clinical applications. The time frame ranged from 2005 to the present, with earlier landmark studies included when needed to clarify mechanistic ideas.

Search terms were organized into concept blocks and combined with Boolean operators. The first block addressed GLP-1 pharmacology and its relevance to periodontal tissues and included terms such as “GLP-1 receptor agonist,” “GLP-1RA,” “semaglutide,” “liraglutide,” “exenatide,” “tirzepatide,” “periodontal tissue,” “gingival tissue,” and “oral tissue.” The second block targeted periodontal and peri-implant disease domains and included “periodontitis,” “periodontal,” “peri-implantitis,” “alveolar bone,” and “gingival inflammation.” The third block addressed mechanistic and regenerative concepts, including “DPP-4,” “oral microbiome,” “PDLSC,” and “osteogenesis.” Search strings were adapted to each database’s syntax.

Reference lists of eligible primary studies, scoping reviews, and narrative reviews were also searched manually to identify additional relevant publications, particularly preclinical and translational studies that may not have been indexed consistently with dental keywords.

Evidence was categorized into five groups: mechanistic and in vitro studies, animal and preclinical studies, observational human studies, interventional clinical studies, and reviews or guidelines for context. Mechanistic and in vitro studies were included when they examined pathways related to gingival inflammation, osteoclastogenesis, osteogenesis, periodontal ligament stem cells, dipeptidyl peptidase-4 (DPP-4) biology, or other host-response mechanisms. Animal and preclinical studies were included when they assessed periodontitis, peri-implant tissues, alveolar bone remodeling, or implant osseointegration in relation to GLP-1 therapies. Observational human studies were included if they reported outcomes on periodontal, peri-implant, metabolic, salivary, or oral adverse effects associated with GLP-1RA exposure. Interventional clinical studies were considered when available, including those on periodontal treatment or implants in GLP-1RA-exposed populations. Reviews, consensus statements, and guidelines supported disease definitions, oral–systemic connections, and methodological context but were not used as primary evidence for estimating effects.

Each included study was evaluated using a structured framework developed by the authors specifically for this review, as no previously published appraisal tool addresses the particular challenges of translational periodontal research on GLP-1RAs. The framework integrates five domains drawn from established principles of translational evidence appraisal, adapted to the specific methodological issues encountered in this field. The first assessment criterion was model relevance, which involved determining whether the study population or experimental model represented diabetic, obese, or metabolically healthy conditions, and whether this aligned with the clinical question being addressed. This was especially crucial because many GLP-1-related effects appear to depend on context and may differ between diabetic and non-diabetic conditions.

The second domain was dose and exposure realism. Studies were assessed for how plausible the drug dose, route, duration, and formulation are in relation to real-world GLP-1RA use. Special attention was given to local delivery systems, nonstandard dosing regimens, and short-duration experiments, which may offer mechanistic insights but are less directly applicable to periodontal practice.

The third domain was periodontal endpoint validity. Studies were evaluated based on whether they used clinically meaningful and methodologically sound outcomes, including periodontal inflammatory indices, radiographic bone measures, histologic variables, osteoclast and osteoblast markers, or validated regenerative readouts. Human studies were also assessed for consistency with current periodontal terminology and outcome definitions.

The fourth domain focused on controlling confounding factors, including baseline glycemic status, diabetes duration, obesity severity, smoking, use of other medications, timing of periodontal treatment, and oral hygiene habits. This domain was emphasized because confounding is a major limitation of current human evidence and often leads to overinterpretation in oral–systemic research.

The fifth domain was translational strength. This assessment evaluated whether a study offered mechanistic plausibility alone, preclinical proof of concept, hypothesis-generating clinical association, or evidence sufficiently strong to influence practice. Using this framework, the review aims to distinguish promising biological signals from evidence that is sufficiently robust to guide periodontal and implant decisions.

## 3. GLP-1 Biology Relevant to Periodontal Tissues

### 3.1. Canonical GLP-1 Signaling

Glucagon-like peptide-1 (GLP-1) is an incretin peptide derived from proglucagon and produced predominantly by intestinal enteroendocrine L-cells, with additional synthesis in pancreatic islet alpha-cells and specific brainstem neurons [28]. The GLP-1 receptor (GLP-1R) is a class B G protein-coupled receptor expressed in multiple tissues, and its activation regulates glucose-dependent insulin secretion, suppresses glucagon secretion, delays gastric emptying, and controls appetite [9]. Native GLP-1 has a very short circulating half-life because it is rapidly degraded by DPP-4, which makes pharmacologic receptor agonism both biologically and clinically significant [28].

From a periodontal standpoint, the traditional GLP-1/DPP-4 axis is significant because it connects metabolic regulation with inflammatory signaling, vascular biology, and tissue turnover [29]. DPP-4 exists in both membrane-bound and soluble forms, and its functions go beyond incretin degradation to include processing immunoregulatory peptides and interacting with immune pathways [30,31,32,33]. This expanded understanding supports the idea that GLP-1-based treatments may affect periodontal tissues through mechanisms beyond glycemic control alone.

GLP-1RAs were developed to address the short half-life of endogenous GLP-1 [34]. Currently available agents vary significantly in molecular structure, half-life, and exposure profile. Short-acting and long-acting compounds exhibit different pharmacokinetic and pharmacodynamic behaviors, which can influence tissue responses, tolerability, and the balance between fasting and postprandial effects [35,36]. This difference is important for periodontal interpretation because sustained receptor activation may have different effects on chronic inflammation and bone remodeling compared to intermittent exposure. Tirzepatide also requires separate consideration because it is a dual glucose-dependent insulinotropic polypeptide (GIP)/GLP-1 receptor agonist rather than a pure GLP-1RA [35].

Table 1 summarizes the key pharmacological characteristics of currently approved GLP-1RAs and the dual GIP/GLP-1 receptor agonist tirzepatide. These agents can be grouped into short-acting compounds (exenatide twice daily, lixisenatide), which produce intermittent receptor activation with pronounced postprandial effects, and long-acting compounds (liraglutide, dulaglutide, semaglutide, extended-release exenatide), which provide sustained receptor stimulation and more consistent effects on fasting glucose, body weight, and systemic inflammation [35,36]. The distinction is periodontally relevant for several reasons. Sustained receptor activation, as achieved with semaglutide and tirzepatide, may produce different effects on chronic inflammatory and bone remodeling pathways than intermittent exposure [9]. In addition, agents differ in their anti-inflammatory profiles: recent evidence suggests that tirzepatide’s dual GIP/GLP-1R agonism may confer additional anti-inflammatory activity through direct actions on GIP receptor-expressing immune cells [37]. Gastrointestinal tolerability also varies across agents, with semaglutide and tirzepatide showing the highest rates of nausea and diarrhea during dose escalation, which has direct implications for oral dryness, hydration, and perioperative management in periodontal care [38]. Most preclinical periodontal evidence is based on liraglutide and exendin-4, whereas clinical exposure is now dominated by semaglutide and tirzepatide.

GLP-1R signaling interacts with pathways involved in systemic low-grade inflammation, oxidative stress, endothelial function, and bone metabolism, all of which are central to the development and progression of periodontitis [8]. Even before considering dental-specific evidence, the canonical incretin pathway offers a mechanistic basis for hypothesizing effects on gingival inflammation, periodontal ligament biology, alveolar bone turnover, and peri-implant tissue responses [30].

### 3.2. Pleiotropic Effects Beyond Glycemia

GLP-1RAs are of interest in periodontology not only because they lower glucose but also because their biological actions extend beyond glycemic control. Current mechanistic reviews consistently describe anti-inflammatory effects, including reducing pro-inflammatory signaling cascades, lowering cytokine production, and modulating immune cell activity [47,48,49,50,51,52]. These actions are directly relevant to periodontal disease, where dysregulation of the host response and chronic cytokine-driven tissue injury are key factors in clinical breakdown [53].

Modulation of oxidative stress is another potential mechanism by which GLP-1-based signaling could protect periodontal tissues. In various non-dental experimental systems, especially vascular and metabolic models, GLP-1-related signaling has been associated with reduced reactive oxygen species production, improved mitochondrial function, and less oxidative damage [49]. Since oxidative stress worsens periodontal inflammation and connective tissue damage [54,55], these findings strengthen the argument for periodontal benefits, particularly in patients with diabetes and obesity who already face a higher oxidative burden [56].

Two additional pleiotropic mechanisms deserve attention in the periodontal context. First, GLP-1R has been shown to be expressed on human platelets, and liraglutide directly attenuates thromboxane-induced platelet aggregation through a GLP-1R-dependent mechanism in obese adults with prediabetes, independently of weight loss or glycemic change [57]. Since platelet activation contributes to vascular inflammation and platelets release growth factors central to bone regeneration, including platelet-derived growth factor and transforming growth factor-beta, this pathway may be relevant to periodontal wound healing and alveolar bone repair, although direct periodontal evidence is lacking. Second, GLP-1 has been shown to promote macrophage polarization toward the anti-inflammatory M2 phenotype via STAT3 activation [58] and modulation of the JNK/STAT3 pathway [59]. This is consistent with the periodontal-specific observation by Sawada et al. that liraglutide shifted gingival macrophages away from an M1-dominant state in a ligature-induced periodontitis model [39], and extends its relevance to obesity and diabetes, where macrophage polarization is characteristically skewed toward a pro-inflammatory M1 phenotype. Together, these platelet- and macrophage-mediated pathways broaden the mechanistic framework for GLP-1RA effects in periodontal tissues beyond the inflammatory cytokine and osteoclastogenesis pathways discussed above.

Bone biology is equally important because alveolar bone loss is the primary structural consequence of periodontitis and a key factor in peri-implant prognosis. Although periodontal evidence remains inconsistent, the wider GLP-1/DPP-4 research supports the idea that incretin-related pathways interact with osteoclast and osteoblast function, bone resorption signals, and inflammatory processes within the bone microenvironment [33]. These diverse actions provide a mechanistic framework for understanding preclinical periodontal studies showing reduced bone loss or improved osseous healing in metabolically compromised conditions.

Another area with translational importance is endothelial and microvascular function. GLP-1RAs have been linked to improved endothelial signaling, reduced endothelial inflammation, and enhanced angiogenic or microvascular integrity in cardiovascular studies [40,60]. Direct evidence from periodontal vascular data remains limited, but these mechanisms are clearly relevant to gingival blood flow, inflammation resolution, and postoperative healing [61]. Practically, the same widespread effects that support cardiorenal benefits may also help improve periodontal and peri-implant wound healing in certain patients, though this remains a hypothesis, awaiting targeted human research.

### 3.3. DPP-4 as the Periodontal-Metabolic Link

In host tissues, DPP-4 (CD26) is a multifunctional serine peptidase that exists in both membrane-bound and soluble forms, with functions in incretin degradation, immune regulation, and inflammatory signaling [62,63]. This dual role in enzymatic activity and immune function makes it especially important in chronic inflammatory conditions such as periodontitis, where local tissue inflammation can affect systemic mediators [64].

Emerging periodontal literature has reinforced this concept by identifying DPP-4-related activity within periodontopathic ecosystems. Experimental work by Ohara-Nemoto and colleagues showed that periodontopathic bacteria express DPP family enzymes and that plaque DPP activity is strongly associated with key anaerobic periodontopathic taxa, including *Porphyromonas* and *Tannerella* species [65]. Earlier work from the same group further demonstrated that bacterial DPP-4 can degrade incretins and alter glucose handling in vivo, providing mechanistic proof of concept that bacterial peptidase activity may carry systemic metabolic consequences [66].

This convergence between host and microbial biology has led to a more refined hypothesis for oral–systemic crosstalk: periodontal dysbiosis may alter incretin signaling through local and potentially systemic DPP-4 or DPP-4-like activity, thereby aggravating glucoregulatory dysfunction and the inflammatory burden in susceptible individuals [8]. Recent scoping reviews have explicitly framed DPP-4 and microbial DPP-4-like activity as candidate mechanisms linking periodontitis with impaired metabolic control, particularly in the setting of diabetes and obesity [8,67].

**Figure 1:**
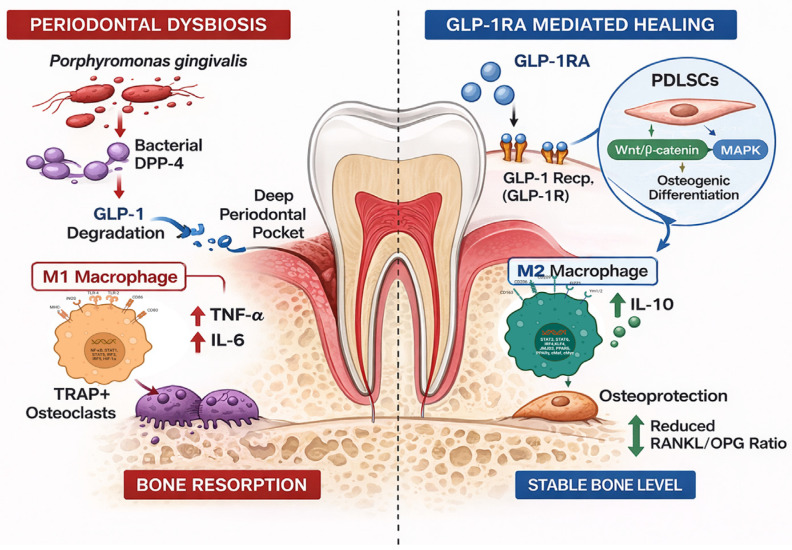
Conceptual model of the GLP-1/DPP-4 axis in periodontal homeostasis and dysbiosis. Experimental and preclinical data suggest that bacterial DPP-like activity, inflammatory signaling, osteoclastogenesis, and altered periodontal ligament stem cell (PDLSC) behavior may interact within periodontal disease. GLP-1RA-related signaling has been associated in preclinical systems with reduced inflammatory activity and preservation of bone-related parameters. The figure summarizes biologically plausible pathways and should not be interpreted as a confirmed sequence in human periodontal tissues.

DPP-4 should therefore be regarded not only as the enzyme that shortens endogenous GLP-1 half-life but also as a candidate mediator at the interface of periodontal inflammation, microbial proteolysis, and systemic metabolic dysregulation (Figure 1). This perspective helps explain why GLP-1 pathway therapeutics may matter in periodontology, even when the observed clinical effects are modest: they act within a biologic network in which periodontal inflammation and incretin physiology may be more closely interconnected than previously assumed.

## 4. Pathobiological Mechanisms in Periodontology

### 4.1. Gingival Inflammation and Immune Modulation

The strongest preclinical evidence linking GLP-1RAs to improvements in periodontal health is the reduction in gingival inflammation. In ligature-induced periodontitis models, liraglutide reduced inflammatory cell infiltration and lowered the expression of gingival inflammatory genes, including tumor necrosis factor-alpha (TNF-α) and inducible nitric oxide synthase, leading to a decrease in tissue inflammation [39]. In the same model, liraglutide also shifted macrophage polarization away from an M1-dominant state without a clear rise in M2 markers, supporting a host-response regulatory effect rather than an antimicrobial action [39].

Across the broader GLP-1RA literature, anti-inflammatory activity is often described as the suppression of nuclear factor kappa B (NF-kappaB)-related signaling and the reduction in cytokines such as IL-1β, IL-6, and TNF-α, with some evidence of increased anti-inflammatory mediators, including IL-10 [68,69]. Although much of this evidence comes from non-dental models, periodontal-focused reviews consistently identify these pathways as biologically plausible and relevant to gingival inflammation [6,7]. More recent mechanistic syntheses also support the idea that GLP-1-based drugs exert anti-inflammatory effects through both metabolic improvement and direct immune or neuroimmune actions, which is important when interpreting periodontal outcomes in patients with diabetes or obesity [37].

An important upstream mechanism that warrants specific attention is the NLRP3 inflammasome. This multi-protein complex serves as a key platform for caspase-1-dependent maturation and secretion of IL-1β and IL-18, both of which are centrally involved in periodontal tissue destruction and alveolar bone resorption [70,71,72]. In periodontitis, NLRP3 inflammasome activation is triggered by periodontopathic bacteria, including *Porphyromonas gingivalis*, and promotes osteoclastic differentiation and alveolar bone loss [71]. Importantly, NLRP3 activity is also upregulated in metabolic conditions such as obesity and diabetes, in which hyperglycemia, dyslipidemia, and oxidative stress serve as additional activating signals, thereby creating a convergence between metabolic and periodontal inflammation [72]. From a GLP-1RA perspective, accumulating evidence demonstrates that GLP-1 and its receptor agonists suppress NLRP3 inflammasome activation through multiple pathways, including inhibition of NF-κB signaling [73], SIRT1-dependent deacetylation [74], and attenuation of reactive oxygen species production [75]. GLP-1-mediated NLRP3 suppression has been demonstrated in perivascular adipose tissue, renal podocytes, and cardiomyocytes under hyperglycemic conditions and represents a biologically plausible mechanism through which GLP-1RAs could reduce inflammasome-driven periodontal tissue injury, particularly in metabolically compromised patients. However, direct evidence of GLP-1RA-mediated suppression of NLRP3 in periodontal tissues is not yet available.

Neutrophil dysregulation is central to periodontitis pathogenesis, but direct periodontal evidence on the effect of GLP-1RAs on neutrophil function remains limited [76]. At present, the rationale is largely translational rather than periodontal-specific: recent mechanistic reviews in chronic wound-healing biology suggest that GLP-1RAs may improve neutrophil antimicrobial function and support reparative immune responses [77]. This is a promising hypothesis for periodontology, but it still requires direct investigation in the gingival and crevicular immune compartments.

Oxidative stress is another plausible mechanism that is increasingly discussed. Recent scoping work in the periodontal GLP-1 field highlights animal evidence suggesting that liraglutide may reduce periodontal oxidative stress, including through nuclear factor erythroid 2-related factor 2 (Nrf2)/HO-1-related signaling in obesity-associated periodontal disease models [8]. Overall, the current evidence supports a model in which GLP-1RA therapy may decrease gingival inflammatory signaling, reduce oxidative injury, and modify immune cell behavior, although the exact role of direct local signaling versus systemic metabolic improvement remains unclear.

### 4.2. Alveolar Bone Metabolism

The relevance of GLP-1RAs to alveolar bone metabolism is clinically significant because bone loss is the defining structural outcome of periodontitis [78] and a key factor in peri-implant prognosis [79]. In preclinical models of periodontitis, liraglutide has been shown to decrease alveolar bone resorption on micro-CT and histology, and to reduce the number of tartrate-resistant acid phosphatase (TRAP)-positive osteoclasts on the alveolar bone surface [39]. In the same study, osteoblast-related alkaline phosphatase activity was not significantly increased, indicating that the dominant effect in that model was suppression of osteoclastogenesis rather than stimulation of osteoblast function [39].

This distinction is important. Much of the available animal evidence indicates that bone preservation occurs through the control of inflammation and decreased resorptive activity, rather than through a strong anabolic response [7]. This pattern makes biological sense because TNF-α and related inflammatory mediators promote osteoclastogenesis, and cytokine suppression via GLP-1RA could, in turn, reduce bone resorption [80]. It also aligns with recent periodontal reviews and studies, which suggest that the strongest current evidence supports bone-preserving effects in inflammatory–metabolic settings rather than confirmed regenerative effects in humans [80,81,82].

The receptor activator of nuclear factor kappa B ligand-osteoprotegerin (RANKL-OPG) axis is highly relevant biologically, but direct periodontal GLP-1RA data on this pathway are limited in the most commonly cited models. Several studies suggest reduced osteoclast activity, as indicated by TRAP staining, decreased inflammation, and less bone loss, rather than by direct measurement of RANKL and OPG [83,84]. This limitation of the literature remains significant and is a key area for future mechanistic research.

Overall, preclinical evidence supports a net bone-preserving effect in periodontitis models, particularly in metabolically compromised settings. The field still requires more standardized bone endpoints and detailed mechanistic measurements to determine whether different GLP-1RAs produce similar osteometabolic effects or whether these responses are molecule-specific and model-dependent.

### 4.3. Periodontal Ligament Stem Cells and Regeneration

The literature on periodontal ligament stem cells (PDLSCs) is one of the most advanced areas in the GLP-1RA-periodontology field, as it goes beyond inflammation control to include regeneration-related biology. Multiple in vitro studies show that exendin-4 can restore or improve osteogenic differentiation of PDLSCs under inflammatory or hyperglycemic conditions, which is especially relevant for diabetic periodontitis and regenerative treatment planning [85,86,87,88].

In an LPS-induced inflammatory PDLSC model, exendin-4 promoted osteogenic differentiation and regulated Wnt and NF-kappaB signaling, supporting a dual anti-inflammatory and pro-osteogenic mechanism [87]. In a high-glucose PDLSC model, exendin-4 likewise improved osteogenic capacity and modulated MAPK and Wnt pathways, thereby increasing its translational relevance for diabetes-related periodontal tissue dysfunction [86]. These studies are especially informative because they focus on the cell population most essential for periodontal regeneration and homeostasis.

Regeneration-oriented preclinical studies have expanded these findings through combined strategies. Stromal-cell-derived factor-1 (SDF-1)/exendin-4 cotreatment has been shown to increase PDLSC proliferation, migration, and osteogenic differentiation in vitro, while also improving periodontal bone regeneration in vivo, as evidenced by micro-CT and histologic indicators of new bone formation [88]. This indicates that GLP-1-related agents might be most effective as parts of bioactive regenerative systems rather than as standalone molecules.

From a translational perspective, these findings support further investigation of GLP-1RA-based adjuncts in guided tissue regeneration, scaffold-based therapies, and biomaterial-assisted periodontal reconstruction. However, the current evidence remains preclinical, and dose selection, release kinetics, local tissue exposure, and compatibility with established regenerative materials will all need careful optimization before clinical use [6,8].

### 4.4. Peri-Implant Tissues and Osseointegration

Peri-implant tissue biology represents a particularly important extension of GLP-1RA research because diabetes impairs osseointegration, bone remodeling, and peri-implant immune regulation [89,90,91]. This creates a strong biologic rationale for investigating whether GLP-1-related therapies can improve implant healing in metabolically compromised hosts. Although evidence is limited, the preclinical data are becoming more consistent.

In diabetic rat implant models, local delivery of exendin-4 through chitosan- poly(lactic-co-glycolic acid) (PLGA) microspheres improved osseointegration and peri-implant bone formation without altering blood glucose levels, an especially important translational observation because it supports a local tissue-level effect independent of systemic glycemic change [92]. The study also illustrates how peri-implant applications may offer a practical translational route for GLP-1-based strategies, particularly through biomaterial-mediated local delivery.

More broadly, recent periodontal and implant-focused reviews describe preclinical evidence that GLP-1RAs can improve peri-implant bone remodeling and osseointegration under diabetic conditions, while also noting that the field remains dominated by animal data and heterogeneous models [93,94]. These reviews highlight a central limitation: implant outcomes have been measured with variable endpoints, model designs, and drug regimens, making direct comparison difficult.

Human evidence in peri-implant settings remains limited and should be interpreted with caution. A retrospective study involving patients with T2D on different hypoglycemic regimens reported variations in peri-implant clinical and radiographic outcomes, including marginal bone loss, and is often cited as an early clinical indicator relevant to GLP-1RA exposure [95]. However, as with any retrospective medication comparison, confounding factors such as indication, baseline metabolic status, concomitant therapy, and treatment history significantly restrict causal conclusions.

Currently, the peri-implant literature supports a promising hypothesis rather than definitive practice changes: GLP-1RA-related pathways may improve implant healing and peri-implant bone preservation in patients with metabolic dysregulation, especially when delivered locally, but robust prospective human studies are still necessary before clinical protocols can be updated.

Table 2 and Table 3 present the existing evidence by summarizing (i) in vitro mechanistic studies and (ii) animal/preclinical in vivo studies relevant to periodontal and peri-implant tissues.

## 5. Clinical Evidence in Humans

### 5.1. Evidence Map

Human evidence regarding GLP-1RAs in periodontology remains limited and should be interpreted cautiously [6,7,8]. The literature is still dominated by mechanistic and preclinical studies, while direct clinical evidence in periodontal care is sparse. Recent reviews centered on periodontal health agree on this point and highlight that, although the translational potential is promising, human data are currently insufficient to draw firm causal conclusions or to inform prescribing practices in periodontal treatment [6,7].

A clear way to organize the available human evidence is to categorize studies into four areas: periodontal outcomes in T2D patients receiving GLP-1RAs, obesity without diabetes, peri-implant outcomes, and comparisons across different antidiabetic treatments. In T2D, direct human data on periodontal outcomes are very limited. The recent narrative review by Ahmad et al. highlighted extensive preclinical research but only limited clinical evidence, with a primary focus on peri-implant outcomes rather than traditional periodontal clinical parameters [6]. Therefore, current claims of periodontal benefits in T2D should be viewed as biologically plausible but not yet clinically confirmed [6,7].

In cases of obesity without diabetes, the evidence is less direct and relates more to incretin axis biology than directly to the effects of GLP-1RAs. Solini et al. [97] observed that severely obese individuals with periodontitis had altered glucoregulatory hormone profiles, including reduced GLP-1 levels, supporting the idea that periodontal inflammation may be linked to impaired incretin function in obesity [97]. Suvan et al. [98] later found that non-surgical periodontal therapy was associated with increased circulating levels of GLP-1 and GIP in both obese and non-obese participants, with a faster rise in GLP-1 among the obese group [98]. These studies provide mechanistic insights but do not prove that GLP-1RA treatment enhances periodontal outcomes in obesity; instead, they highlight the bidirectional connection between oral health and metabolism, which drives interest in this area.

Peri-implant outcomes currently offer the most direct human clinical indicator, but the evidence remains observational. The key study is the retrospective cohort study by Shi et al., which compared implants in patients with T2D receiving different hypoglycemic regimens and evaluated radiographic marginal bone loss alongside peri-implant clinical parameters [95]. This study is significant because it provides a comparative clinical framework and suggests that the type of antidiabetic regimen may affect peri-implant hard-tissue outcomes; however, it remains susceptible to confounding by indication, baseline metabolic differences, and treatment history effects inherent to retrospective designs. Therefore, it should be viewed as hypothesis-generating rather than definitive proof of a GLP-1RA-specific peri-implant benefit.

Comparative evidence against metformin–, insulin–, or sodium–glucose cotransporter 2 (SGLT2) inhibitor-based regimens remains a significant challenge. Existing retrospective implant studies compare different hypoglycemic medication groups but lack the methodological controls needed for causal inference, and there are no strong periodontal comparative-effectiveness studies with standardized endpoints directly evaluating GLP-1RAs against SGLT2 inhibitors or insulin-based strategies in routine care [6]. This limitation is especially important because modern antidiabetic therapies may share anti-inflammatory and metabolic effects [99,100,101,102,103], making it difficult to make class-specific periodontal claims without direct human comparisons.

Taken together, the human evidence map supports a balanced conclusion. The strongest current signal is not a demonstrated direct periodontal therapeutic effect of GLP-1RAs, but rather a possible oral–systemic interaction in which metabolic therapy may influence periodontal and peri-implant outcomes. The field is advancing, but for now, the literature on human research remains preliminary and should be interpreted as hypothesis-generating.

### 5.2. Clinical Endpoints to Extract

For a clinically meaningful and comparable synthesis, human studies in this area should be evaluated using a predefined set of periodontal, peri-implant, metabolic, and follow-up variables. This is especially important because the current evidence base is sparse and heterogeneous, and inconsistency in outcomes can easily lead to overinterpretation of weak signals [6,8].

In studies addressing periodontal outcomes, core clinical parameters should include probing depth (PD), clinical attachment level (CAL), bleeding on probing (BOP), and plaque index (PI), ideally recorded with clearly described probing protocols and examiner calibration. These remain the most interpretable indicators of inflammatory burden, tissue destruction, and treatment response in periodontology and should be reported at both baseline and follow-up to enable longitudinal interpretation [104].

Radiographic bone outcomes should also be extracted systematically. In periodontal studies, this includes radiographic bone loss or change in bone level over time, whereas in peri-implant studies, the preferred hard-tissue outcome is marginal bone loss (MBL), ideally reported separately for mesial and distal surfaces when available. The retrospective study by Shi et al. [95] illustrates why radiographic metrics matter: the principal between-group signal was detected in peri-implant MBL rather than in soft-tissue variables such as BOP or probing depth [95]. This distinction is important because it suggests that any early human signal may be more readily detectable in bone remodeling than in peri-implant soft-tissue inflammation.

Tooth loss and implant survival (or implant success, when explicitly defined) should be extracted whenever follow-up duration allows, even if these are not primary endpoints. These outcomes are less sensitive to short-term biological changes [105,106,107,108], but they are clinically decisive and enhance the translational value of the evidence map. In the current GLP-1RA literature, such endpoints are rarely available with adequate follow-up, and this should be acknowledged as a limitation rather than interpreted as evidence of no effect [6].

Metabolic variables are indispensable for interpreting every human study because they are potential mediators rather than mere background descriptors. At minimum, studies should report glycated hemoglobin (HbA1c) and body mass index (BMI), and ideally also weight change, duration of diabetes, and, when available, inflammatory biomarkers such as CRP, IL-6, or TNF-α. Without these variables, it becomes difficult to determine whether a periodontal or peri-implant finding reflects direct tissue-level GLP-1 signaling or indirect improvement in systemic metabolic control [7,8].

Finally, follow-up duration and periodontal treatment exposure must be reported explicitly. Studies should state clearly whether patients received supportive periodontal therapy, non-surgical treatment, surgery, implant maintenance, or no standardized periodontal intervention during the observation period. This is not a minor methodological detail; it is a major determinant of endpoint behavior and one of the most common reasons clinical associations become uninterpretable in oral–systemic research [7].

### 5.3. Major Confounders

Currently, the main risks are not only small sample sizes or observational designs but also the tendency to attribute periodontal or peri-implant differences to GLP-1RA exposure without properly accounting for metabolic and behavioral factors that significantly impact oral outcomes. Reviews in this area have already emphasized that apparent periodontal benefits are likely influenced heavily by indirect systemic effects and study design limitations [6,8].

Baseline glycemic control is one of the most important confounders. Patients initiating or receiving GLP-1RAs often differ from comparator groups in HbA1c, glycemic variability, and overall intensity of diabetes management [109,110,111]. Because glycemic control strongly influences periodontal inflammation, wound repair, and peri-implant bone remodeling [112,113,114], failure to adjust for baseline HbA1c and subsequent glycemic change can create the false impression of a direct periodontal drug effect. This is especially relevant in retrospective comparisons with insulin or metformin, where prescribing patterns often reflect disease severity and treatment history rather than random allocation [115,116].

The duration of diabetes should be considered separately from HbA1c. Two patients with similar HbA1c values may differ markedly in cumulative metabolic burden, vascular dysfunction, accumulation of advanced glycation end-products, and impairment of the host response, depending on whether one has longstanding disease and the other has recent-onset disease [117,118]. This distinction is particularly important for peri-implant outcomes and regenerative endpoints, where chronic metabolic exposure may affect bone and soft-tissue healing independently of current glucose control [119,120].

The severity of obesity is another major confounder and, in some cases, a mediator. GLP-1RAs are increasingly prescribed for obesity, and weight loss itself can modify systemic inflammatory tone, adipokine signaling, and insulin sensitivity [121,122]. Any observed improvement in oral health during GLP-1RA therapy may therefore reflect changes in adiposity rather than a direct pharmacologic effect on periodontal tissues. This is one reason why obesity-only incretin axis studies are informative but cannot be interpreted as treatment-effect studies for periodontitis. Future studies should report baseline BMI, weight trajectory, and, ideally, central adiposity measures to improve interpretability.

Smoking remains a core periodontal confounder and must be modeled explicitly. It affects inflammatory phenotype, vascular response, healing capacity, and bone remodeling [123,124,125], and may also correlate with medication adherence and healthcare utilization [126,127,128,129,130,131,132]. In small observational cohorts, even modest imbalances in smoking status can materially distort periodontal or peri-implant comparisons [129]. Smoking should therefore not be treated as a simple descriptive variable but incorporated into adjusted models or stratified analyses whenever possible.

Concomitant medication use deserves special attention. Metformin, insulin, SGLT2 inhibitors, statins, antihypertensives, and anti-inflammatory drugs may all directly or indirectly influence periodontal or peri-implant outcomes [133,134]. In the current human literature, comparator groups are often defined by hypoglycemic regimen, but medication combinations and background therapies are not always adequately described. This is a major limitation because observed differences may reflect combination effects, differential disease severity, or prescribing bias rather than GLP-1RA exposure alone. The absence of head-to-head periodontal endpoint studies against SGLT2 inhibitors is a particularly important challenge, given the overlapping systemic anti-inflammatory and metabolic effects of these drug classes.

The timing of periodontal treatment and oral hygiene behavior is frequently underreported but remains a central confounder in oral–systemic studies. A patient who recently received scaling and root planing, entered supportive care, or improved plaque control at home may show lower BOP and probing depths regardless of changes in metabolic medication [135,136]. Conversely, poor adherence to maintenance can obscure systemic improvement [137,138]. Human studies should therefore report the timing of periodontal treatment relative to GLP-1RA initiation, maintenance intervals, plaque-control measures, and oral hygiene reinforcement protocols. Without this information, changes in clinical endpoints cannot be confidently attributed to drug exposure.

Nutritional changes during GLP-1RA therapy are an often-overlooked confounder and should be explicitly considered. GLP-1RAs typically cause appetite suppression, changes in meal patterns, and, in some patients, reduced protein intake or dehydration linked to gastrointestinal side effects [38]. These changes can affect oral inflammatory burden, salivary function, postoperative recovery, and healing ability. Therefore, nutritional changes can mediate both benefits and risks, and ignoring this variable can significantly distort interpretation. Recent reviews in the oral and periodontal GLP-1RA literature have already highlighted that many observed oral benefits should be viewed as preliminary and possibly indirect, which aligns with this confounding framework [7].

Adherence and proper dose escalation remain crucial. Typically, GLP-1RAs are introduced gradually, with early discontinuation or dose reduction due to tolerability issues being common in real-world settings [139,140,141]. Studies that only categorize participants as “GLP-1 users” without detailed data on duration, dose, persistence, or recent initiation risk significant misclassification of exposure. This is especially concerning for periodontal and peri-implant outcomes, which usually develop over months rather than weeks. To enhance causal inference, future clinical research should report drug-specific exposure duration, maintenance dose, escalation stages, and adherence metrics.

Taken together, these confounders support a central interpretive principle: in human studies, GLP-1RA exposure should be understood as part of a broader immunometabolic treatment context rather than as an isolated periodontal intervention. That principle is essential both for avoiding overstatement and for designing clinically meaningful future research.

### 5.4. Bottom-Line Interpretation

The current human evidence supports a cautious but clinically relevant conclusion. GLP-1RAs may be relevant to periodontal and peri-implant health within a broader metabolic context, but the strongest signal at present is one of risk modulation and tissue preservation rather than a stand-alone periodontal therapeutic effect. This interpretation is consistent with the broader literature, which shows strong mechanistic plausibility and encouraging preclinical findings, but very limited direct human periodontal outcome data [6,7,8].

The evidence is most compelling in metabolically dysregulated populations, especially patients with T2D and obesity, where GLP-1RAs might plausibly enhance periodontal or peri-implant outcomes through combined effects on glycemia, adiposity, systemic inflammation, and possibly tissue-level inflammatory and osteogenic pathways [142,143,144]. However, the available human clinical data are not yet sufficient to confidently distinguish these indirect systemic effects from direct periodontal pharmacology. The single retrospective peri-implant study is clinically interesting and hypothesis-generating but cannot establish causality or specify prescribing implications for implant care.

There is likewise insufficient evidence at present to support formal periodontal prescribing recommendations or to position GLP-1RAs as host-modulatory periodontal agents in routine practice. Within periodontal medicine, these drugs are better viewed as systemic therapies with possible oral implications that may alter risk profile and treatment response in selected patients.

Therefore, GLP-1RAs are currently best understood as part of a broader oral–systemic therapeutic approach where metabolic optimization may support periodontal stability and peri-implant preservation, while their direct periodontal effects still need to be proven through well-designed human studies with standardized endpoints and thorough control of confounding factors.

Table 4 summarizes human studies with GLP-1RA (or GLP-1 drug) exposure and oral outcomes. Table 5 presents human incretin axis studies relevant to periodontal disease or periodontal therapy that do not involve GLP-1RA exposure (contextual mechanistic clinical evidence).

## 6. GLP-1RAs and Oral Adverse Effects

### 6.1. Xerostomia, Salivary Hypofunction, and Salivary Changes

The current literature on GLP-1RAs and oral health is weighted toward potential anti-inflammatory and osteometabolic benefits rather than adverse oral symptoms. Xerostomia and salivary hypofunction, in particular, remain less systematically discussed. This omission is clinically important because salivary function is central to plaque control, mucosal integrity, and long-term periodontal maintenance [146,147,148,149], especially in patients with diabetes and obesity, who may already be predisposed to oral dryness and impaired healing [150,151,152].

Recent mechanistic narrative work has proposed a plausible biological basis for semaglutide-related oral dryness. In a 2025 review focused on GLP-1 receptor signaling and oral dysfunction, Barac and Roganovic summarized evidence suggesting that GLP-1 receptor activity in salivary tissues may be influenced by agonist-specific signaling patterns, including differences in cAMP- and beta-arrestin-related pathways, with semaglutide discussed as a candidate for prolonged receptor stimulation and possible receptor desensitization or internalization within salivary gland contexts [150]. Importantly, the authors present this as a mechanistic hypothesis rather than an established causal pathway, which is appropriate given the current level of evidence.

The same review also noted disproportionality signals for oral adverse events, including dry mouth, in pharmacovigilance datasets, with semaglutide reported more frequently in some analyses than other GLP-1RAs, while emphasizing that spontaneous-report systems cannot establish incidence or causality [150]. This caution is supported by a 2025 analysis of otolaryngologic adverse events in the Food and Drug Administration (FDA) Adverse Event Reporting System (FAERS), which identified significant reporting signals for dry mouth with semaglutide and distinct event patterns for other GLP-1RAs, including liraglutide and exenatide, across different symptom clusters [153]. Such datasets are useful for signal detection and hypothesis generation, but they remain inherently vulnerable to reporting bias, stimulated reporting, and confounding by indication or comorbidity.

At the clinical level, direct evidence remains limited but noteworthy. A 2023 case series described three patients with semaglutide-associated hyposalivation or xerostomia, with symptom onset during therapy, objective reduction in salivary flow, and improvement after symptomatic management, including drug discontinuation in some cases [145]. The authors appropriately presented this as an initial signal requiring prospective validation, which remains the correct interpretation given the very small sample and non-comparative design.

For periodontology, the relevance of xerostomia and salivary hypofunction extends beyond comfort. Saliva maintains oral homeostasis through mechanical clearance, buffering, antimicrobial activity, lubrication, and protection of hard and soft tissues [154]. Reduced salivary flow is associated with greater plaque retention, higher risk of caries and demineralization, increased mucosal irritation or infection, and more difficulty maintaining oral hygiene, all of which can complicate periodontal maintenance and peri-implant care [155,156,157]. In practical terms, even a modest medication-associated increase in oral dryness may amplify disease risk in patients who already have diabetes-related xerostomia, polypharmacy, or limited self-care capacity.

This issue should nevertheless be framed with restraint. First, oral dryness has not been established as a uniform class effect across all GLP-1RAs, and available data suggest heterogeneity among agents rather than a single pattern [153]. Second, xerostomia in GLP-1RA users is likely multifactorial, with plausible contributions from reduced oral intake, altered thirst behavior, dehydration, concomitant medication use, diabetes itself, and gastrointestinal adverse effects in addition to any direct salivary gland signaling effects [158]. Third, the current evidence base is dominated by mechanistic inference, pharmacovigilance analysis, and case-level reports rather than by prospective oral medicine or periodontal cohorts [150].

**Figure 2:**
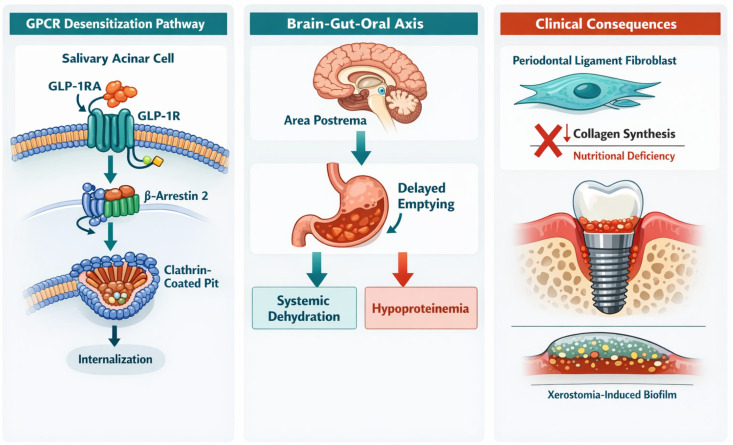
Proposed pathways by which GLP-1RA therapy may influence oral dryness and healing-related oral conditions. Three mechanistic domains are illustrated. Left panel: the GPCR desensitization pathway, in which prolonged GLP-1RA stimulation of the GLP-1R on salivary acinar cells may lead to β-arrestin 2 recruitment, clathrin-mediated receptor internalization, and reduced salivary secretory function; this mechanism remains hypothetical and may differ across agents. Center panel: the brain–gut–oral axis, in which GLP-1RA-mediated activation of the area postrema and delayed gastric emptying may contribute to systemic dehydration and hypoproteinemia, particularly during dose escalation. Right panel: potential clinical consequences, including impaired collagen synthesis by periodontal ligament fibroblasts due to nutritional deficiency and xerostomia-induced biofilm accumulation affecting periodontal and peri-implant tissues. These pathways may act independently or in combination and should be interpreted as a conceptual model rather than a confirmed tissue-level mechanism. Abbreviations: GLP-1RA, glucagon-like peptide-1 receptor agonist; GLP-1R, glucagon-like peptide-1 receptor; GPCR, G protein-coupled receptor.

Xerostomia and salivary hypofunction should therefore be regarded as emerging oral safety considerations in GLP-1RA therapy, with current concern most clearly centered on semaglutide-related signals, but with insufficient evidence to define incidence, causality, or molecule-specific risk with precision (Figure 2). In periodontal practice, this supports a pragmatic approach: monitor symptoms of dryness, salivary function, plaque accumulation, and mucosal tolerance during maintenance care [159], while avoiding overstatement of causal drug effects until prospective comparative data become available. The evidence derives mainly from mechanistic reviews, pharmacovigilance analyses, and case-level observations rather than from prospective periodontal cohorts.

### 6.2. Indirect Oral Effects of Gastrointestinal Adverse Events

The oral relevance of GLP-1RA therapy is not confined to direct salivary gland or mucosal effects. A second clinically important pathway is indirect, mediated through gastrointestinal adverse events that can alter hydration status, nutritional intake, and adherence to postoperative instructions [150]. This is especially pertinent in periodontology and implant dentistry, where short-term tissue healing and long-term maintenance depend on adequate hydration, nutrition, and patient compliance [159].

Nausea, vomiting, and diarrhea are among the most common adverse reactions reported for semaglutide and tirzepatide in prescribing information, and both the U.S. FDA and the EMA (European Medicines Agency) explicitly note that gastrointestinal reactions may lead to dehydration or volume depletion, especially during treatment initiation and dose escalation [41,42,43,44,45,46]. This is directly relevant to oral care because dehydration can worsen oral dryness, reduce salivary flow, and increase plaque retention and mucosal discomfort, thereby complicating periodontal maintenance and peri-implant hygiene in susceptible patients [156,160].

Dietary change represents a second major indirect pathway. GLP-1RAs reduce appetite and slow gastrointestinal transit [161], which may improve weight and glycemic outcomes but can also compromise nutrient intake if diet quality is not actively managed [162]. Recent nutrition-focused work suggests that reduced intake, nausea, and food aversion during GLP-1RA therapy may contribute to suboptimal protein and micronutrient intake, raising concern about nutritional deficiencies in real-world use [163,164,165]. Although this literature is not periodontal-specific, it is clearly relevant to periodontal and implant healing because protein sufficiency and micronutrient adequacy are fundamental to collagen synthesis, immune competence, and tissue repair [166,167,168].

From a periodontal standpoint, these nutritional effects should be viewed as plausible modifiers of wound healing rather than as established GLP-1RA-specific oral toxicities. The current evidence warrants concern about reduced intake and nutritional imbalance during therapy, but most studies focus on obesity or nutrition, and very few assess periodontal surgery, regenerative procedures, or peri-implant healing directly in relation to dietary changes during GLP-1RA use [163]. Maintaining this distinction is important for scientific balance.

These indirect effects also have practical implications for postoperative care and adherence. Early GLP-1RA dose escalation is often the period during which nausea and reduced oral intake are most prominent [169,170], and this may interfere with hydration, analgesic tolerance, oral hygiene, and adherence to dietary instructions after periodontal surgery or implant placement. In such cases, delayed recovery or greater discomfort may reflect a combination of medication-related gastrointestinal symptoms and reduced oral intake rather than surgical factors alone (inference based on prescribing information and oral healing requirements) [157].

A balanced clinical interpretation is therefore that gastrointestinal adverse events associated with GLP-1RAs may have meaningful secondary consequences for periodontal and peri-implant care through dehydration- and nutrition-related pathways. These effects are likely to matter most during treatment initiation or dose escalation and in patients with pre-existing xerostomia, diabetes-related healing impairment, or complex postoperative needs. At present, the evidence supports heightened clinical awareness and monitoring, but not the adoption of class-wide assumptions about oral complications in all GLP-1RA users. These considerations are clinically plausible but remain inferential in the absence of prospective oral-healing studies in GLP-1RA-exposed patients.

### 6.3. Medication Management Considerations in Periodontal Practice

As GLP-1RAs become more common among patients with T2D and obesity, periodontal practice must adapt by incorporating these medications into routine risk assessment, treatment planning, and maintenance care. The current evidence does not support GLP-1RAs as periodontal therapies in their own right, but it does support their relevance as systemic agents that may influence inflammatory status, hydration, nutrition, and perioperative recovery [52,170]. Medication management in periodontal care should therefore emphasize structured history-taking, interdisciplinary communication, and prevention of avoidable complications during active therapy and maintenance [171].

A first priority is to refine medical history protocols so that GLP-1RA exposure is documented more precisely than by a simple yes/no medication entry. Clinically relevant details include the specific agent (for example, semaglutide, liraglutide, or tirzepatide), therapeutic indication (T2D, obesity, or both), route of administration, and treatment phase, particularly whether the patient is in early dose escalation or stable maintenance. This matters because gastrointestinal intolerance, appetite suppression, and the risk of dehydration are often more pronounced during initiation and titration [171], which, in turn, may affect postoperative tolerance and home-based care behavior [41,42,43,44,45,46]. Recording treatment duration and any recent dose changes is likewise helpful, as these variables influence the interpretation of oral symptoms and healing trajectories over time [150].

The indication for therapy should be documented explicitly, as it helps contextualize periodontal risk and confounding factors. A patient receiving a GLP-1RA for longstanding T2D with vascular complications presents a different periodontal and peri-implant risk profile from a patient using the same drug for obesity without diabetes [6]. In the former, glycemic history, diabetes duration, and polypharmacy may dominate healing outcomes; in the latter, weight-loss trajectory, diet quality, and xerostomia/salivary hypofunction risk may be more influential [6,8].

Coordination with the treating physician or endocrinologist is advisable in complex cases, particularly when periodontal surgery, implant placement, advanced regeneration, or prolonged healing is expected. This is not because GLP-1RAs are contraindicated in periodontal care, but because patients receiving these drugs may have fluctuating nutritional intake, active dose escalation, or broader metabolic instability [162,163] that influences perioperative resilience. Interprofessional communication is especially valuable when there is uncertainty regarding glycemic control, recent medication changes, recurrent gastrointestinal intolerance, or concomitant use of multiple glucose-lowering agents such as insulin plus a GLP-1RA or a GLP-1RA plus an SGLT2 inhibitor. In this context, physician input helps the dental team distinguish local postoperative problems from systemic medication-related contributors and supports safer, more individualized care.

Hydration and nutrition deserve particular attention during periodontal surgery and supportive periodontal therapy. In practical terms, GLP-1RA-related nausea, early satiety, and reduced fluid intake may indirectly worsen oral dryness, increase plaque accumulation, and reduce tolerance of postoperative hygiene instructions [150]. These effects matter most during active surgical healing, when tissue repair depends on adequate hydration, sufficient protein intake, and consistent self-care [167]. The available evidence does not justify assuming that all GLP-1RA users will heal poorly, but it does support a precautionary approach in patients who report persistent gastrointestinal symptoms or reduced oral intake.

A balanced clinical position is therefore to regard GLP-1RA therapy as a relevant contextual factor in periodontal management, not as a contraindication, and not yet as a periodontal adjunctive treatment. Its practical value lies in careful documentation of the agent and treatment phase, anticipation of hydration- and nutrition-related challenges, and coordination of care when systemic complexity is high. This approach is conservative, clinically useful, and aligned with the present evidence base, which supports vigilance and individualized management, while direct periodontal prescribing implications remain unproven.

## 7. Implications for Periodontal and Implant Practice

### 7.1. Risk Stratification in the Periodontal Chart

In contemporary periodontal practice, the use of GLP-1RA should be recorded as a contextual clinical factor rather than simply an entry on the medication list. GLP-1RA exposure usually reflects an underlying metabolic profile, most commonly T2D, obesity, or both, and these conditions are themselves major determinants of periodontal and peri-implant risk [6]. A chart entry that records only “semaglutide” or “tirzepatide” is therefore insufficient for interpreting periodontal risk; the documentation framework described in Section 6.3 should be applied [41,42,43,44,45,46,171].

**Figure 3:**
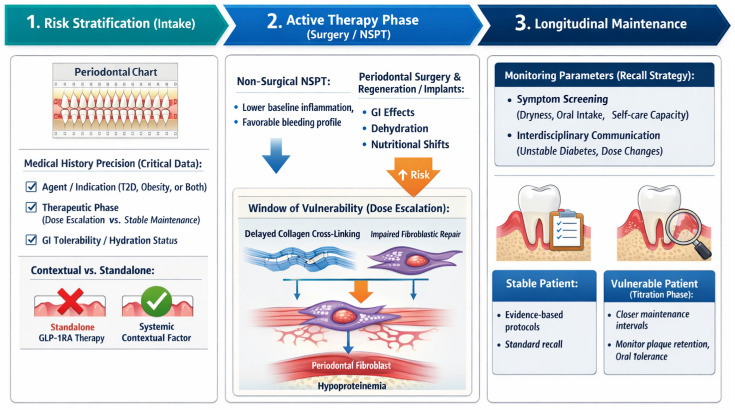
Proposed clinical framework for periodontal and implant patients receiving GLP-1RA therapy. During treatment initiation or dose escalation, clinicians may consider closer monitoring for gastrointestinal intolerance, dehydration, xerostomia, and reduced oral intake. In stable maintenance phases, routine periodontal and implant care should remain guided by established protocols, with GLP-1RA use incorporated as a contextual factor in risk assessment, maintenance planning, and interdisciplinary communication.

From a periodontal medicine perspective, GLP-1RA use is best situated within the broader domains of systemic disease control and obesity or T2D management. This framing helps clinicians interpret periodontal findings in context, especially when inflammation, healing, or maintenance behavior changes over time. A patient receiving GLP-1RA therapy may show improved metabolic control and lower inflammatory burden, but the same patient may also develop xerostomia/salivary hypofunction, reduced oral intake, or fluctuating self-care capacity, all of which can influence plaque control and maintenance stability [150]. Documenting these variables in the periodontal chart improves longitudinal interpretation and supports more individualized recall planning. Effective management requires a longitudinal perspective that accounts for the pharmacodynamic transition from dose titration to steady-state maintenance (Figure 3). This approach is consistent with a periodontal medicine model in which systemic treatment changes are incorporated into risk assessment rather than treated as incidental medical background [172].

### 7.2. Non-Surgical Periodontal Therapy

For non-surgical periodontal therapy, the most plausible interaction with GLP-1RA use is through reduction in background inflammatory burden rather than a direct periodontal pharmacologic effect. Current reviews support the view that GLP-1RAs may improve the systemic inflammatory and metabolic context in which periodontal treatment is delivered, particularly in patients with T2D or obesity, but they also emphasize that direct human periodontal evidence remains limited [6,7]. In practice, clinicians may observe more favorable bleeding profiles or a lower overall inflammatory tone after metabolic improvement, but such changes should not be automatically attributed to a direct gingival drug effect without considering glycemic changes, weight loss, and oral hygiene behavior [8].

One practical implication is that closer maintenance intervals may be useful initially in some GLP-1RA-treated patients, particularly during periods of active metabolic change or recent medication titration. This is not because GLP-1RA therapy is inherently destabilizing, but because the early treatment phase may involve nausea, dehydration, xerostomia/salivary hypofunction, and altered eating patterns that can temporarily compromise plaque control and mucosal comfort [41,42,43,44,45,46]. Shorter early recalls may help the clinical team monitor plaque accumulation, bleeding trends, symptom burden, and adherence to home care while the systemic regimen is stabilizing.

Monitoring changes in BOP and PD after metabolic improvement is also clinically informative, but interpretation should remain conservative. If periodontal inflammation decreases after GLP-1RA initiation, this may reflect improved glycemic control, reduced adipose-driven inflammation, better health behavior, or stronger adherence to periodontal care rather than a direct tissue-level GLP-1 effect [8]. Preserving that distinction in chart notes and clinical discussion is central to a balanced reading of the current evidence.

### 7.3. Periodontal Surgery and Regeneration

In periodontal surgery and regenerative therapy, GLP-1RA use may offer potential advantages, but these are currently indirect and context dependent. The most plausible benefits include lower systemic inflammatory burden and improved glycemic control in metabolically dysregulated patients, both of which are relevant to wound healing and postoperative stability [6]. Preclinical and translational reviews support this biological rationale, but current human surgical data are insufficient to establish procedure-specific benefit [6]. GLP-1RA therapy should therefore be viewed as a potentially favorable systemic background factor rather than as an independent determinant of surgical prognosis.

Potential risks in the perioperative period are more immediately actionable. The gastrointestinal adverse effects and dehydration risks, as described in Section 6.2, may aggravate oral dryness, reduce tolerance to hygiene instructions, and interfere with early healing, particularly during dose escalation [41,42,43,44,45,46]. These risks warrant routine screening before surgery and during follow-up.

A practical perioperative approach should therefore focus on hydration, nutrition, and communication. In patients who report significant gastrointestinal symptoms, the surgical team should reinforce hydration and postoperative intake planning and consider closer follow-up during the early healing period. When systemic complexity is high, such as unstable diabetes, recent GLP-1RA dose escalation, recurrent gastrointestinal intolerance, or use of multiple glucose-lowering agents, coordination with the treating physician or endocrinologist is appropriate to improve perioperative safety and to help interpret healing problems. This is a precautionary interdisciplinary strategy, not a recommendation for routine alteration of systemic GLP-1RA treatment from the periodontal side.

### 7.4. Implant Dentistry

In implant dentistry, the preclinical evidence, as discussed in Section 4.4, supports the biological plausibility of improved osseointegration and peri-implant bone remodeling with GLP-1RA exposure, but these findings should not yet be translated into changes in clinical protocols [8].

Peri-implant maintenance in GLP-1RA users should therefore remain anchored in established evidence-based implant protocols, with added attention to the same contextual variables discussed for periodontology: metabolic control, xerostomia/salivary hypofunction, hydration status, and adherence to home care and recall. The currently available human peri-implant signal comes from a single retrospective study that reported lower marginal bone loss in GLP-1 drug users than in some other hypoglycemic regimen groups, but did not show clear superiority in soft-tissue inflammatory parameters and was limited by study design and attrition [95].

At present, GLP-1RA use should inform risk assessment and maintenance planning rather than implant protocol selection. The evidence is encouraging regarding osseointegration hypotheses and peri-implant bone preservation in metabolically compromised patients, but it is not yet strong enough to justify GLP-1RA-specific changes in implant timing, loading, surgical technique, or maintenance interval beyond individualized clinical judgment.

## 8. Controversies and Limitations

### 8.1. Is the Effect Direct or Indirect?

The central controversy in this field is whether the apparent periodontal and peri-implant benefits associated with GLP-1RAs reflect direct receptor-mediated actions in oral tissues or indirect effects driven by systemic metabolic improvement. This question is not merely mechanistic; it also determines whether GLP-1RAs should be regarded as genuine host-modulatory candidates in periodontology or primarily as systemic therapies that improve the biologic context in which periodontal disease develops and is treated.

At present, the strongest support for direct periodontal effects comes from preclinical and in vitro studies showing anti-inflammatory signaling, reduced osteoclastogenesis, and improved osteogenic differentiation in periodontal-related cells and models. These observations support biological plausibility, but they do not fully disentangle local receptor-mediated effects from concurrent changes in systemic inflammation, glycemia, and weight in whole-animal models [39,86,96]. By contrast, human evidence is sparse and observational, making it even harder to separate tissue-level actions from broader immunometabolic improvement. For now, the most defensible interpretation is that both pathways are plausible, but the indirect systemic pathway is better supported in humans [6].

Direct and indirect effects should therefore be treated less as a binary opposition than as points along a continuum. In metabolically dysregulated patients, periodontal improvement may arise from multiple mechanisms, but the current evidence base does not permit confident estimation of their relative contributions.

### 8.2. Drug-Class Effect or Molecule-Specific Effect

A second major limitation of the current literature is the tendency to treat “GLP-1RAs” as a uniform class with interchangeable periodontal implications. That assumption is unlikely to be fully accurate. Exenatide, liraglutide, semaglutide, and tirzepatide differ in structure, half-life, receptor pharmacology, dosing interval, and, in the case of tirzepatide, receptor profile, because tirzepatide is a dual GIP/GLP-1 receptor agonist rather than a pure GLP-1RA [41,42,43,44,45,46].

These differences matter for periodontal interpretation. Exposure profile and receptor signaling dynamics may influence inflammatory pathways, appetite-related effects, dehydration risk, and tissue-level responses [173]. The emerging literature on oral adverse effects also suggests heterogeneity across molecules, with semaglutide showing stronger pharmacovigilance signals for dry mouth in some datasets, whereas other agents display different adverse-event patterns [150]. In parallel, much of the preclinical periodontal and peri-implant evidence centers on liraglutide, exendin-4, or exenatide-based systems, while real-world human exposure is increasingly dominated by semaglutide and tirzepatide [8]. This translational mismatch should be acknowledged explicitly.

Future studies should therefore avoid broad class-level conclusions when the underlying data come from only one or two molecules. A more rigorous approach is to report molecule-specific evidence first and then discuss whether a class-level inference is justified.

### 8.3. Population Specificity

Population specificity is another underdeveloped area and a major source of uncertainty. The biological and clinical significance of GLP-1RA use in periodontology is likely to differ across metabolic phenotypes, yet many discussions in the literature implicitly generalize findings from T2D to obesity without diabetes, or from well-controlled disease to poorly controlled disease.

In patients with T2D, especially those with longstanding or poorly controlled disease, any periodontal effect of GLP-1RA therapy is more plausibly mediated by improvements in glycemia, the inflammatory burden, and metabolic stability [6]. In obesity without diabetes, the current human evidence is largely indirect, focusing on incretin axis perturbation and the response to periodontal treatment rather than on the clinical outcomes of GLP-1RA therapy itself [97,98]. These studies are informative, but they should not be treated as therapeutic evidence of periodontal benefit.

Population heterogeneity also includes older adults and patients with polypharmacy, who may be more vulnerable to dehydration, xerostomia, nutritional issues, and medication interactions [174]. These concerns are especially relevant in periodontal surgery and implant care, yet they are seldom examined in current studies. Similarly, smokers and patients with severe periodontitis phenotypes might respond differently because smoking and disease severity significantly influence inflammation, vascular response, and healing [104]. Without stratified analysis, the field risks averaging effects that lack clinical relevance for high-risk groups. An important question is not only whether GLP-1RAs are helpful, but also who is most likely to experience improvements in periodontal and peri-implant health.

### 8.4. Endpoint Heterogeneity

Endpoint heterogeneity is a major methodological weakness in the current evidence base and is one reason which explains why the literature remains difficult to compare across studies. Periodontal and peri-implant investigations often use different definitions, different combinations of clinical and radiographic outcomes, and different follow-up intervals. Some emphasize inflammatory soft-tissue variables, whereas others report mainly radiographic bone findings, making cross-study synthesis difficult even when the overall direction of effect appears favorable [6].

This problem is compounded by inconsistent alignment with contemporary periodontal terminology. The 2018 World Workshop introduced a staging and grading framework that is now the standard for case definition and risk characterization, yet many oral–systemic studies still use older terminology or fail to report disease stage and grade clearly [104]. For a topic as sensitive to confounding as GLP-1RA use, this is especially limiting because stage and grade directly affect prognosis, treatment complexity, and interpretation of systemic modifiers.

Future clinical studies should align with modern periodontal definitions and report the core clinical and radiographic endpoints outlined in Section 5.2, together with clear follow-up timing and documentation of periodontal treatment received. Standardization will be essential if the field is to move beyond hypothesis generation.

### 8.5. Evidence Imbalance

The most obvious limitation in this field is the imbalance between abundant preclinical evidence and limited human clinical data. Robust periodontal intervention trials are still lacking [8], and the available human studies—dominated by indirect incretin axis investigations and retrospective designs—remain vulnerable to the confounders discussed in Section 5.3 [6].

## 9. Future Research

### 9.1. Priority Clinical Study Designs

Progress in this field will depend on moving beyond mechanistic plausibility and retrospective signals toward clinically interpretable human study designs. The most immediately feasible approach is a prospective cohort model in which patients receiving GLP-1 receptor agonists (GLP-1RAs) are followed longitudinally during standardized periodontal therapy and supportive care, with predefined periodontal and metabolic endpoints. Such a design would not eliminate confounding, but it would substantially improve existing retrospective evidence by allowing baseline characterization, repeated measures, and explicit documentation of medication exposure, dose escalation, glycemic changes, and the timing of periodontal treatment.

Randomized controlled trials focused on diabetic periodontal populations should also be considered, although feasibility will vary by setting. The most realistic RCTs are likely to take the form of adjunctive or comparative-care models among patients already eligible for systemic metabolic treatment, rather than studies in which periodontal teams direct diabetes prescribing. For example, periodontal outcomes could be assessed prospectively in collaboration with endocrinology or obesity medicine clinics where antidiabetic regimens are initiated according to medical criteria and periodontal measurements are incorporated as predefined secondary outcomes. Even if fully drug-randomized periodontal trials prove difficult, pragmatic randomized designs addressing periodontal treatment timing, maintenance intensity, or perioperative support in GLP-1RA users could still generate valuable evidence.

Comparative-effectiveness research is especially important and remains underdeveloped. The main clinical question is not simply whether GLP-1RAs are associated with better periodontal outcomes than no treatment, but whether they differ meaningfully from other common metabolic regimens, particularly SGLT2 inhibitors, metformin-based combinations, and insulin-based strategies. This is where the field could become most clinically informative. Because modern antidiabetic therapies share overlapping anti-inflammatory and metabolic effects, comparative studies with robust adjustment for indication bias and baseline disease severity are essential if periodontal medicine is to make drug-specific rather than generic metabolic control inferences.

### 9.2. Core Outcome Set Proposal

A major obstacle to synthesis in the current literature is heterogeneity in outcomes. Future studies should adopt a standardized core outcome framework spanning the periodontal, peri-implant, systemic, oral function, and safety domains. Such a framework would improve comparability across studies and reduce the risk that apparently favorable findings are driven by selective endpoint reporting.

For periodontitis-focused studies, the minimum periodontal set should follow the endpoint framework defined in Section 5.2 (PD, CAL, BOP, PI, and radiographic bone variables), interpreted within the 2018 staging and grading framework. In peri-implant studies, marginal bone loss should be paired with probing-based inflammatory outcomes and suppuration, as hard- and soft-tissue signals may diverge [95].

Systemic outcomes should be mandatory in any GLP-1RA periodontal study, as they are likely mediators of the observed oral effects. At minimum, studies should report HbA1c and weight or body mass index, and ideally also high-sensitivity C-reactive protein as a pragmatic inflammatory marker. Without these variables, interpretation of direct versus indirect effects remains weak even when periodontal endpoints appear to improve.

Oral function outcomes should also be standardized, particularly as oral adverse effects become more prevalent in the literature. Salivary flow measurement and validated xerostomia scales would make future safety analyses considerably more informative than isolated symptom reporting alone. This is especially relevant in maintenance and perioperative settings, where dryness and reduced oral intake may affect plaque control, comfort, and adherence [145,150].

Finally, safety outcomes should be captured systematically rather than narratively. Gastrointestinal adverse effects, hydration-related indicators, and basic nutritional markers or intake proxies should be included whenever possible, particularly in surgical and implant cohorts. These variables are not peripheral; they may directly affect healing behavior and postoperative recovery and are likely to explain part of the real-world variability observed in GLP-1RA users [164].

### 9.3. Translational Work

The next phase of translational research should prioritize direct human tissue evidence. A major deficiency is the limited characterization of GLP-1 receptor-related signaling in human gingival and periodontal ligament tissues under clinically relevant conditions. Receptor-expression studies in gingival and periodontal ligament tissues, ideally stratified by diabetes status, obesity, and periodontal stage or grade, would help determine whether the tissue-level targets proposed in preclinical work are consistently present and biologically meaningful in patients [8].

Single-cell transcriptomic approaches in periodontal and peri-implant tissues could significantly advance the field by identifying which cell populations are most responsive to incretin-related signaling and how these responses change within inflammatory and hyperglycemic microenvironments. Such research would be especially useful for clarifying the roles of immune cells, fibroblasts, endothelial cells, and progenitor populations in any GLP-1RA-related periodontal phenotype. The PDLSC literature already indicates pathway-specific effects involving MAPK, Wnt/beta-catenin, and inflammatory signaling, but these findings need validation in more complex human tissue systems [86,87].

Oral microbiome research should also move beyond taxonomy toward functional profiling. The DPP-4 link is one of the most biologically interesting aspects of this field, but human studies remain limited. Future translational designs should combine detailed periodontal phenotyping with microbiome profiling and direct measurement of oral DPP-4 or DPP-4-like activity in saliva, gingival crevicular fluid, and subgingival plaque, ideally alongside incretin-related systemic biomarkers. This would permit a more rigorous test of the proposed pathway linking dysbiosis, peptide degradation, and metabolic inflammation.

Local delivery platforms represent another promising area. Preclinical research using exendin-4-loaded microspheres in diabetic implant models has already demonstrated proof of concept for site-specific osteogenic support without significant systemic glycemic effects [88]. This is highly relevant for periodontal regeneration and peri-implant defect management. Future research should build on this by using hydrogels, injectable carriers, and membrane-associated platforms for periodontal and peri-implant applications, while also improving dose accuracy and release kinetics for clinical translation.

### 9.4. Implementation Science

Implementation science is likely to determine whether this topic becomes clinically useful or remains mechanistically interesting but operationally disconnected from practice. A first priority is the development of practical methods for integrating GLP-1RA-related information into periodontal risk-assessment models. This includes not only documenting the medication name but also recording the indication, dose phase, tolerability, hydration status, xerostomia symptoms, and concomitant metabolic therapies in a structured format that can be incorporated into periodontal charting and recall planning.

A second priority is the development of interdisciplinary care pathways linking periodontology with endocrinology and obesity medicine. The clinical issues in this field are inherently shared: glycemic control, obesity management, hydration, nutrition, medication tolerability, and oral inflammatory burden all intersect. Implementation studies should therefore evaluate referral triggers, communication templates, and co-management workflows for patients undergoing periodontal surgery, implant therapy, or intensive maintenance while receiving GLP-1RAs. This is especially relevant in older adults, patients with polypharmacy, and those with unstable metabolic disease, in whom isolated dental or medical management is less likely to capture the full risk profile [174].

More broadly, implementation-focused research can help the field avoid a common translational failure point: generating compelling biologic hypotheses without producing usable clinical processes. In the context of GLP-1RAs and periodontology, the most effective near-term contribution may be standardized clinical documentation, coordinated care pathways, and prospective practice-based data collection. These steps would strengthen both patient care and the underlying evidence base while larger comparative and interventional studies continue to mature.

## 10. Conclusions

GLP-1 receptor agonists are increasingly being discussed as potentially relevant to periodontal biology within the context of metabolic disease. Preclinical studies consistently suggest anti-inflammatory, osteometabolic, and regenerative effects in periodontal and peri-implant tissues, while the emerging role of the DPP-4 axis in host–microbe interactions adds further mechanistic plausibility. However, human evidence remains limited and mainly observational. Therefore, the most balanced view currently is that any periodontal benefits linked to GLP-1RA therapy are more likely due to improved systemic metabolic control rather than definitively proven direct effects on periodontal tissues.

Clinically, GLP-1RA therapy should be regarded as a factor that may influence periodontal risk and healing capacity rather than as a therapeutic modality for periodontal disease itself. In this context, medication-related variables, including the specific agent, indication, treatment stage, and patient tolerability, should be considered during risk assessment and maintenance planning. Particular attention is warranted for xerostomia/salivary hypofunction, hydration status, and nutritional adequacy, especially during dose escalation.

Further progress will depend on prospective cohort studies and comparative-effectiveness investigations with standardized periodontal and peri-implant endpoints, translational studies evaluating GLP-1 receptor expression and DPP-4 activity in human periodontal tissues, and implementation research that promotes collaboration among periodontology, endocrinology, and obesity medicine. Until more solid evidence appears, both premature treatment decisions and unjustified dismissals should be avoided, as the proposed link remains biologically plausible and clinically significant.

## Figures and Tables

**Table 1:** Pharmacological characteristics of approved GLP-1RAs and tirzepatide relevant to periodontal interpretation.

Agent	Structural Basis	Receptor Target	Half-Life	Dosing	Relative Weight Loss	Relative GI Adverse Effects	Preclinical Periodontal Evidence
Exenatide (twice daily)	Exendin-4	GLP-1R	2.4 h	Twice daily, SC	+	Moderate (vomiting higher)	Exendin-4 basis for PDLSC and implant studies
Exenatide ER	Exendin-4	GLP-1R	~2 weeks (microsphere)	Once weekly, SC	++	Lower than twice daily	—
Lixisenatide	Exendin-4	GLP-1R	3 h	Once daily, SC	+	Moderate	—
Liraglutide	Human GLP-1 analogue	GLP-1R	13 h	Once daily, SC	++	Moderate	Periodontitis models [39,40]
Dulaglutide	Human GLP-1 analogue (Fc fusion)	GLP-1R	~5 days	Once weekly, SC	++	Lower	—
Semaglutide (SC)	Human GLP-1 analogue	GLP-1R	~7 days	Once weekly, SC	+++	Higher (nausea, diarrhea)	—
Semaglutide (oral)	Human GLP-1 analogue	GLP-1R	~7 days	Once daily, oral	++ to +++	Higher	—
Tirzepatide	Synthetic dual agonist	GIP/GLP-1R	~5 days	Once weekly, SC	++++	Higher (nausea, diarrhea)	—

Abbreviations: GLP-1R, glucagon-like peptide-1 receptor; GIP, glucose-dependent insulinotropic polypeptide; SC, subcutaneous; ER, extended release; PDLSC, periodontal ligament stem cell; GI, gastrointestinal. Relative weight loss and GI adverse effects are approximate comparative rankings based on clinical trial data and should not be interpreted as absolute quantitative comparisons: +, low; ++, moderate; +++, high; ++++, very high. Preclinical periodontal evidence is summarized from available experimental studies [1,35,36,41,42,43,44,45,46].

**Table 2:** In vitro mechanistic evidence on GLP-1/GLP-1RA signaling in periodontal-relevant systems.

Study	Cell System/ Model	Stress Context	Intervention	Key Endpoints	Main Finding	Translational Note
Guo et al., 2018 [85]	Human PDLSCs	High glucose	Exendin-4	Proliferation; ALP; osteogenic differentiation markers	Exendin-4 mitigated high-glucose–related inhibition of PDLSC osteogenic differentiation	Supports plausibility in diabetic microenvironment
Liu et al., 2019 [87]	Human PDLSCs	LPS-induced inflammatory stress	Exendin-4	ALP; mineralization; Runx2/Osx; NF-κB; Wnt signaling	Exendin-4 enhanced osteogenic differentiation while modulating Wnt and NF-κB signaling under inflammatory stress	Pathway-resolved; dose/exposure realism should be judged vs. clinical concentrations
Wang et al., 2023 [86]	Human PDLSCs	High glucose	Exendin-4	Osteogenic markers; MAPK and Wnt readouts	Exendin-4 alleviated high-glucose-induced osteogenic inhibition with MAPK and Wnt pathway involvement	Replicates high-glucose concept with different mechanistic emphasis
Liang et al., 2021 [88]	Human PDLSCs	Regenerative signaling context	SDF-1 + exendin-4	Proliferation; migration; osteogenic differentiation; osteoclastogenesis-related signals	Co-therapy enhanced PDLSC recruitment/osteogenesis and supported a regenerative concept	Links in vitro effects to in vivo defect repair
Ohara-Nemoto et al., 2017 [66]	Periodontopathic bacterial enzyme activity (in vitro assays)	Proteolysis of incretin substrates	Bacterial DPP-4 (periodontopathic)	Incretin degradation; glucose modulation (mechanistic)	Periodontopathic bacterial DPP-4 degraded incretins and was linked to altered glycemic readouts in experimental settings	Supports the DPP-4 host–microbe connection hypothesis (mechanistic)

Abbreviations: PDLSC, periodontal ligament stem cell; ALP, alkaline phosphatase; LPS, lipopolysaccharide; Runx2, runt-related transcription factor 2; Osx, osterix; NF-κB, nuclear factor kappa B; Wnt, Wingless/Integrated signaling pathway; MAPK, mitogen-activated protein kinase; SDF-1, stromal-cell-derived factor-1; DPP-4, dipeptidyl peptidase-4; GLP-1, glucagon-like peptide-1; GLP-1RA, glucagon-like peptide-1 receptor agonist.

**Table 3:** Animal/preclinical in vivo evidence on GLP-1RA and periodontal/peri-implant outcomes.

Study	Model	Metabolic Context	Intervention (Route/Duration)	Primary Periodontal/Implant Endpoints	Key Finding	Key Caveats
Sawada et al., 2020 [39]	Ligature-induced periodontitis (rat)	Non-diabetic	Liraglutide, systemic; 2 weeks	Micro-CT alveolar bone loss; gingival inflammatory markers; macrophage phenotype; TRAP	Liraglutide reduced gingival inflammation and alveolar bone loss, with fewer TRAP+ osteoclasts	Systemic exposure; difficult to separate direct vs. indirect effects
Yang et al., 2022 [96]	Diabetes–periodontitis comorbidity (rat)	Diabetic	Liraglutide, systemic; ~4 weeks	Alveolar bone microstructure; inflammatory cytokines; RANKL/OPG; Runx2/ALP	Liraglutide improved periodontal damage and bone microarchitecture and reduced RANKL/OPG ratio with increased osteogenic markers	Diabetes induction variability; confounding by glycemic improvement
Liang et al., 2021 [88]	Periodontal bone defect model (rat)	Not specified as diabetic	EX-4 (systemic) + topical SDF-1	Micro-CT defect fill; histology; endogenous cell recruitment; osteoclastogenesis	Co-therapy promoted periodontal bone regeneration and modulated remodeling indices	Combined intervention; not GLP-1RA-only effect
Shi et al., 2022 [92]	Dental implant osseointegration (T2D rat)	Diabetic	Local EX-4-loaded chitosan–PLGA microspheres	BIC/BV/TV; peri-implant micro-CT; osseointegration indices	Local exendin-4 delivery improved impaired osseointegration and peri-implant bone formation without relying on systemic glycemic change	Local delivery system; translation depends on dosing, release kinetics, and biomaterial compatibility

Abbreviations: GLP-1RA, glucagon-like peptide-1 receptor agonist; micro-CT, micro-computed tomography; TRAP, tartrate-resistant acid phosphatase; RANKL, receptor activator of nuclear factor kappa B ligand; OPG, osteoprotegerin; Runx2, runt-related transcription factor 2; ALP, alkaline phosphatase; EX-4, exendin-4; SDF-1, stromal-cell-derived factor-1; T2D, type 2 diabetes; PLGA, poly(lactic-co-glycolic acid); BIC, bone–implant contact; BV/TV, bone volume fraction.

**Table 4:** Human studies with GLP-1RA exposure and oral outcomes (periodontal/peri-implant/adverse oral effects).

Study (Year)/Design	Population	Exposure/Comparator	Oral Outcomes	Main Finding	Key Limitations
Shi et al. (2021) [95]; retrospective cohort	T2D implant patients; clinical records-based cohort	GLP-1 drugs in antidiabetic regimen (agent/dose NR) vs. other hypoglycemic medication groups (e.g., metformin/insulin-based regimens)	Peri-implant MBL and clinical parameters; case definitions NR	Signal for lower MBL in GLP-1 drug users vs. some comparators (hypothesis-generating)	Confounding by indication; exposure misclassification; limited soft-tissue endpoints; treatment history heterogeneity; HbA1c reported but other metabolic covariates NR; periodontal/maintenance details NR; limited covariate adjustment
Mawardi et al. (2023) [145]; case series	Mixed adults	Semaglutide (dose and duration variable; NR); no comparator	Hyposalivation/xerostomia (salivary flow and symptoms); periodontal outcomes not primary	Hyposalivation temporally associated with semaglutide in selected cases	Small sample; no control group; reporting bias; causality not established; dental/periodontal treatment context NR

Abbreviations: GLP-1RA, glucagon-like peptide-1 receptor agonist; T2D, type 2 diabetes; MBL, marginal bone loss; HbA1c, hemoglobin A1c; NR, not reported.

**Table 5:** Human incretin axis studies relevant to periodontitis/periodontal therapy (no GLP-1RA exposure; context only).

Study (Year)/Design	Population	Exposure/Periodontal Characterization	Key Endpoints; Follow-Up	Key Finding	Interpretation and Limitations
Solini et al. (2019) [97]; cross-sectional	Severely obese adults (diabetes status NR/variable by cohort)	Naturalistic comparison: periodontitis vs. no/less periodontitis; periodontal status assessed, staging/grading NR	Glucoregulatory hormones including GLP-1 and related peptides; cross-sectional	Periodontitis associated with altered glucoregulatory hormone profile (including lower GLP-1 signal)	Interpretation: Supports oral–metabolic linkage; not treatment evidence for GLP-1RAs. Limitations: Cross-sectional; residual confounding (diet, meds, inflammation); periodontal definitions heterogeneous.
Suvan et al. (2021) [98]; cohort (before–after periodontal therapy)	Obese and non-obese adults	Non-surgical periodontal treatment; periodontal indices assessed, staging/grading NR	Circulating GLP-1 and GIP; metabolic covariates; ≈6 months	Periodontal therapy associated with increased incretin levels (earlier GLP-1 rise in obese participants)	Interpretation: Contextual mechanistic clinical evidence; does not test GLP-1RA effects. Limitations: Non-randomized; lifestyle co-interventions possible; confounding; limited generalizability.

Abbreviations: GLP-1, glucagon-like peptide-1; GLP-1RA, glucagon-like peptide-1 receptor agonist; GIP, glucose-dependent insulinotropic polypeptide; NR, not reported.

## Data Availability

No new data were created or analyzed in this study.

## Abbreviations

The following abbreviations are used in this manuscript:

ALP	alkaline phosphatase
BIC	bone–implant contact
BMI	body mass index
BOP	bleeding on probing
BV/TV	bone volume fraction
CAL	clinical attachment level
CD26	cluster of differentiation 26
cAMP	cyclic adenosine monophosphate
CRP	C-reactive protein
DPP-4	dipeptidyl peptidase-4
EMA	European Medicines Agency
EX-4	exendin-4
FAERS	FDA Adverse Event Reporting System
FDA	Food and Drug Administration
GIP	glucose-dependent insulinotropic polypeptide
GLP-1	glucagon-like peptide-1
GLP-1R	glucagon-like peptide-1 receptor
GLP-1RA	glucagon-like peptide-1 receptor agonist
HbA1c	hemoglobin A1c
HG	high glucose
HO-1	heme oxygenase-1
ICMJE	International Committee of Medical Journal Editors
IL-1β	interleukin-1 beta
IL-6	interleukin-6
IL-10	interleukin-10
LPS	lipopolysaccharide
M1	classically activated macrophage phenotype
M2	alternatively activated macrophage phenotype
MAPK	mitogen-activated protein kinase
MBL	marginal bone loss
MEDLINE	Medical Literature Analysis and Retrieval System Online
micro-CT	micro-computed tomography
NF-kappaB	nuclear factor kappa B
NR	not reported
Nrf2	nuclear factor erythroid 2-related factor 2
OPG	osteoprotegerin
Osx	Osterix
PD	probing depth
PDLSC	periodontal ligament stem cell
PI	plaque index
PLGA	poly(lactic-co-glycolic acid)
RANKL	receptor activator of nuclear factor kappa B ligand
RCT	randomized controlled trial
Runx2	runt-related transcription factor 2
SANRA	Scale for the Assessment of Narrative Review Articles
SDF-1	stromal-cell-derived factor-1
SGLT2	sodium–glucose cotransporter 2
T2D	type 2 diabetes
TNF-α	tumor necrosis factor-alpha
TRAP	tartrate-resistant acid phosphatase
WHO	World Health Organization
Wnt	Wingless/Integrated signaling pathway
